# ONC213: a novel strategy to resensitize resistant AML cells to venetoclax through induction of mitochondrial stress

**DOI:** 10.1186/s13046-024-03267-6

**Published:** 2025-01-09

**Authors:** Jenna L. Carter, Yongwei Su, Eman T. Al-Antary, Jianlei Zhao, Xinan Qiao, Guan Wang, Holly Edwards, Lisa Polin, Juiwanna Kushner, Sijana H. Dzinic, Kathryn White, Steven A. Buck, Maik Hüttemann, Joshua E. Allen, Varun V. Prabhu, Jay Yang, Jeffrey W. Taub, Yubin Ge

**Affiliations:** 1 Cancer Biology Graduate Program, Wayne State University School of Medicine, Detroit, MI 48201 USA; 2 MD/PhD Program, Wayne State University School of Medicine, Detroit, MI 48201 USA; 3 Department of Oncology, Wayne State University School of Medicine, Detroit, MI 48201 USA; 4 Molecular Therapeutics Program, Barbara Ann Karmanos Cancer Institute, Wayne State University School of Medicine, Detroit, MI 48201 USA; 5 Division of Pediatric Hematology/Oncology, Children’s Hospital of Michigan, Detroit, MI 48201 USA; 6 Department of Pediatrics, Central Michigan University College of Medicine, Mt. Pleasant, MI 48859 USA; 7 National Engineering Laboratory for AIDS Vaccine, School of Life Sciences, Jilin University, Changchun, China; 8 Department of Pediatrics, Wayne State University School of Medicine, Detroit, MI 48201 USA; 9 Center for Molecular Medicine and Genetics, Wayne State University School of Medicine, Detroit, MI 48201 USA; 10 Chimerix, Inc, Durham, NC 27713 USA

**Keywords:** Acute myeloid leukemia, Venetoclax, Azacitidine, ONC213

## Abstract

**Background:**

Venetoclax + azacitidine is a frontline treatment for older adult acute myeloid leukemia (AML) patients and a salvage therapy for relapsed/refractory patients who have been treated with intensive chemotherapy. While this is an important treatment option, many patients fail to achieve complete remission and of those that do, majority relapse. Leukemia stem cells (LSCs) are believed to be responsible for AML relapse and can be targeted through oxidative phosphorylation reduction. We previously reported that ONC213 disrupts oxidative phosphorylation and decreases Mcl-1 protein, which play a key role in venetoclax resistance. Here we investigated the antileukemic activity and underlying molecular mechanism of the combination of ONC213 + venetoclax against AML cells.

**Methods:**

Flow cytometry was used to determine drug-induced apoptosis. Protein level changes were determined by western blot. An AML cell line-derived xenograft mouse model was used to determine the effects of ONC213 + venetoclax on survival. A patient-derived xenograft (PDX) mouse model was used to determine drug effects on CD45+/CD34+/CD38-/CD123 + cells. Colony formation assays were used to assess drug effects on AML progenitor cells. Mcl-1 and Bax/Bak knockdown and Mcl-1 overexpression were used to confirm their role in the mechanism of action. The effect of ONC213 + venetoclax on mitochondrial respiration was determined using a Seahorse bioanalyzer.

**Results:**

ONC213 + venetoclax synergistically kills AML cells, including those resistant to venetoclax alone as well as venetoclax + azacitidine. The combination significantly reduced colony formation capacity of primary AML progenitors compared to the control and either treatment alone. Further, the combination prolonged survival in an AML cell line-derived xenograft model and significantly decreased LSCs in an AML PDX model.

**Conclusions:**

ONC213 can resensitize VEN + AZA-resistant AML cells to venetoclax therapy and target LSCs ex vivo and in vivo.

**Supplementary Information:**

The online version contains supplementary material available at 10.1186/s13046-024-03267-6.

## Background

Acute myeloid leukemia (AML) is an aggressive form of acute leukemia that has a sharp rise in incidence with increasing age and is the most common form of acute leukemia in adults [1]. The standard care for AML, which is an intensive regimen of cytarabine and anthracycline-based chemotherapy, remained stagnant for over five decades despite a 5-year overall survival (OS) rate of only 30%. Even with recent increases in available therapies, 5-year survival rates remain low at about 40% for adults younger than 65 years and only 6% for adults who are 65 years and older [2]. Age is an independent prognostic factor in AML which continues to rise as a clinical challenge as the median age of onset of AML has been steadily rising over the years [3].

AML cells are reliant on antiapoptotic proteins such as Bcl-2 and Mcl-1 for survival [4–7]. Inhibition of these proteins has demonstrated success in the development of new therapies [8]. Venetoclax, a selective Bcl-2 inhibitor, was approved by the US FDA in 2018 to be used in combination with low-dose cytarabine (LDAC) or hypomethylating agents (HMAs) in patients who are 75 years or older, or who cannot tolerate intensive chemotherapy (IC). Venetoclax + azacitidine has become a new standard treatment option for many AML patients due to its superior efficacy compared to HMA monotherapy or venetoclax + LDAC and improved tolerability compared with IC [9–12]. Venetoclax + HMA is also now routinely used as salvage therapy for patients who are treated with upfront IC [13–15]. Though this treatment regimen provides an important therapeutic option for this population, majority of patients who initially respond to venetoclax + azacitidine will relapse within 18 months and many patients will fail to achieve complete remission [10]. Additionally, failure from venetoclax and HMA therapy results in highly aggressive and refractory disease with a median overall survival of just 2.4 months [16].

Another major contributor to relapse in AML is thought to be due to a small subpopulation of leukemia stem cells (LSCs) [17–22]. LSCs are capable of self-renewal, have proliferative capacity, and differentiate into AML bulk cells [20, 23]. A high number of LSCs at the time of diagnosis is associated with poor survival outcomes [24]. Investigations have found that LSCs can be targeted through both induction of oxidative stress and reduction of oxidative phosphorylation through inhibition of mitochondrial translation [25, 26].

Previously, we demonstrated that the imipridone, ONC213, suppressed mitochondrial function through mitochondrial stress induction in AML cells [27]. Induction of integrated stress response (ISR) via ONC213 leads to disruption of AML cell metabolism and reduction in Mcl-1, a key protein in venetoclax resistance. ONC213 mitochondrial stress induction also leads to apoptosis in leukemia progenitor cells and LSCs while having low toxicity to normal hematopoietic cells and had promising efficacy as a monotherapy in in vivo studies. Given these promising early monotherapy results, here we investigate the use of ONC213 in combination with venetoclax to address multiple clinical challenges with resistance to venetoclax and azacitidine as well as persistence of LSCs.

## Methods

### Mice

Eight-week-old immunocompromised triple transgenic NSG-SGM3 female mice (NSGS, JAX#103062; non-obese diabetic scid gamma (NOD.Cg-Prkdc^scid^ Il2rg^tm1Wjl^ Tg(CMV-IL3, CSF2, KITLG)1Eav/MloySzJ; Jackson Laboratory, Bar Harbor ME, USA) were used for the leukemia xenograft models. All mice were provided food and water *ad libitum*, given supportive fluids and supplements as needed, and housed within an AAALAC-accredited animal facility with 24/7 veterinary care. All in vivo experiments involving these mice were approved by the Institutional Animal Care and Use Committee at Wayne State University. For all trials, mice were monitored daily for weight changes, signs of leukemia, or signs of toxicity during the duration of treatment; treatment was halted at the first signs of leukemia (hindleg weakness, > 15% weight loss, metastatic spread to internal organs), and they were thereon monitored twice daily for progressing signs of leukemia. Mice were sacrificed based on severity of disease-induced symptoms.

### Patient derived xenograft model

A Patient Derived Xenograft (PDX) model, J000106565, was purchased from Jackson Laboratory. This relapsed model is positive for *FLT3*-ITD, *FLT3*-TKD, and *NPM1* mutations, classified as AML M4/M5, and the patient had undergone induction chemotherapy with consolidation high dose cytarabine and allogenic hematopoietic stem cell transplant. J000106565 cells were passaged in NSGS female mice, as previously described [27]. Cells were injected into the tail-vein of NSGS female mice (1.0 × 10^6^ cells/mouse). Engraftment was verified on day 22 or 18, as indicated, by flow cytometry for hCD45 + cells in the peripheral blood of 3 randomly selected mice. For the venetoclax + azacitidine trial, the mice were randomized into one of four groups (5 mice/group). Starting on day 23, mice were treated with either venetoclax (25 mg/kg, p.o., daily), azacitidine (5 mg/kg, i.v., every four days), venetoclax + azacitidine, or vehicle control. Upon euthanasia, spleens from venetoclax + azacitidine treated mice were harvested, and leukemia cells were isolated from the tissues. Isolated AML cells were used in other studies as outlined below and maintained in culture as described under Clinical Samples. For the ONC213 + venetoclax trial, mice were randomized into one of four groups (5 mice/group). Mice were treated starting on day 19 with either venetoclax (25 mg/kg, p.o., daily), ONC213 (75 mg/kg, p.o., daily), ONC213 + venetoclax, or vehicle control.

### MV4-11 xenograft model

MV4-11 cells were injected into the tail-vein of NSGS female mice (1.0 × 10^6^ cells/mouse, *n* = 20) and randomized into four treatment groups (*n* = 5 per group). Mice were treated with ONC213 (75 mg/kg, p.o., daily; or 180 mg/kg, p.o., every five days), venetoclax (25 mg/kg, p.o., daily; or 25 mg/kg, p.o., for three days followed by 2-day holiday), ONC213 + venetoclax (75 mg/kg ONC213 + 25 mg/kg venetoclax [daily] or 180 mg/kg ONC213 + 25 mg/kg venetoclax [spaced]), or vehicle control starting day 3 after cell injection.

### Clinical samples

Diagnostic blast samples were obtained from the First Hospital of Jilin University. Written informed consent was provided, according to the Declaration of Helsinki. This study was approved by the Human Ethics Committee of The First Hospital of Jilin University (Ethical code # 2019 − 128). Clinical samples were screened for gene mutations by PCR amplification and automated DNA sequencing; the samples were also screened for fusion genes by real-time RT-PCR, as described previously [28, 29]. Patient characteristics are shown in Supplementary Table S1.

Diagnostic blast samples were purified by standard Ficoll-Hypaque density centrifugation, then maintained in culture in RPMI 1640/20% FBS (Thermo Fisher Scientific., Waltham, MA, USA) supplemented with ITS solution (Sigma-Aldrich, St. Louis, MO, USA) and 20% supernatant of the 5637 bladder cancer cell line [as a source of granulocyte-macrophage colony-stimulating factor [30]]. Normal human CD34 + hematopoietic cells were purchased from Novo Biotechnology (Beijing, China).

### Cell lines

MV4-11 and THP-1 cells were purchased from the American Type Culture Collection (Manassas, VA, USA). OCI-AML3 and ML-2 cells were purchased from the German Collection of Microorganisms and Cell Cultures (Braunschweig, Germany). MOLM-13 was purchased from AddexBio (San Diego, CA, USA). The cell lines were authenticated in 2017 at the Genomics Core at Karmanos Cancer Institute using the PowerPlex^®^ 16 System from Promega (Madison, WI, USA). Monthly mycoplasma testing was performed using the PCR method [31]. AML cell lines were maintained as previously described [28, 32]. Establishment of the ML-2 and MV4-11 venetoclax + azacitidine resistant cell lines were previously described [33]. MOLM-13/VEN + AZA-R cells were generated here using the same method as described in [33]. These cells are maintained in the presence of 1500 nM VEN + 4500 nM AZA. Venetoclax resistant cells, MOLM-13/VEN-R and MV4-11/VEN-R, were generated by culturing parental cells in the presence of stepwise increasing concentrations of venetoclax for approximately 12 months. The final concentration of venetoclax was based on the clinical C_max_ of 2 µM [34].

### Drugs

ONC213 was provided by Chimerix, Inc. (Durham, NC, USA). Venetoclax (Venclexta^®^) was purchased from AbMole Bioscience Inc. (Houston, TX). Azacitidine was purchased from Selleck Chemicals (Houston, TX, USA).

### Annexin V- fluorescein isothiocyanate (FITC)/ propidium iodide (PI) staining and flow cytometry analysis

AML cells were treated with indicated drugs for as long as 72 h, and the same volume of treated cells underwent Annexin V-FITC/PI staining (Beckman Coulter, Brea, CA, USA). Samples were analyzed by flow cytometry, as previously described [35, 36]. All experiments using the AML cell lines were performed three times in triplicate independently, while those using primary patient samples were preformed once in triplicate due to limited sample. Apoptotic events are shown as the mean percentage of Annexin V+/PI- (early apoptotic) and Annexin V+/PI+ (late apoptotic and/or dead) cells ± the SEM from one representative experiment. The combination index (CI) was calculated using CompuSyn software (Combosyn Inc., Paramus, NJ, USA) to determine synergy. CI < 1, CI = 1, and CI > 1 indicate synergistic, additive, and antagonistic effects, respectively [37].

### Western blot analysis

Western blots were performed as previously described [38]. Whole-cell lysates were subjected to SDS-PAGE, electrophoretically transferred onto polyvinylidene difluoride membranes (Thermo Fisher Inc., Rockford, IL, USA), and immunoblotted with antibodies. Immunoreactive proteins were visualized using the Odyssey Infrared Imaging System (Li-Cor, Lincoln, NE, USA). Antibodies directed towards PARP, Mcl-1, Bcl-2, Bax, Bcl-xL (Proteintech, Chicago, IL, USA), β-actin (Sigma-Aldrich), Bim, cleaved (cf.) caspase 3, p-eIF2α, eIF2α, and ATF4 (Cell Signaling Technologies, Danvers, MA, USA) were used for Western blot analysis.

### Mitochondrial respiration

Cellular Mito Stress Test (CMST) was conducted using a Seahorse XFe24 flux analyzer (Agilent Technologies, Santa Clara, CA, USA) as previously described [33]. AML cells were treated with ONC213, venetoclax, or the combination for 4–16 h prior to seeding into cell-tak-coated plates (100,000 cells/well for AML cell lines and 400,000 cells/well for J000106565 cells). CMST parameters: oligomycin 1 µM; FCCP 1.5 µM for MV4-11, THP-1, and MV4-11/VEN + AZA-R cells and 0.5 µM for JAX00106565 cells; and rotenone + antimycin A 0.5 µM each. Basal respiration, maximal respiration, spare respiratory capacity (SRC), and OCR associated with ATP production were calculated per the manufacturer’s instructions.

### Assessment of mitochondrial membrane potential

AML cells were treated with ONC213, venetoclax, or the combination for 4–8 h and then resuspended in fresh media containing 1 µM JC-1 (Solarbio Science & Technology, Beijing, China) and incubated for 15 min at 37 °C. The samples were washed, resuspended in PBS, and then assessed by flow cytometry analyses.

### Isolation of human CD34 + cells

CD34 MicroBead kit (130-046-702, Miltenyi Biotec, Auburn, CA) was used to isolate human CD34 + cells from mouse spleen cell suspensions, according to the manufacturer’s instructions.

### Mcl-1 overexpression, Mcl-1 knockdown and Bak/Bax double knockdown

Lentivirus production and transduction were carried out as previously described [39]. The pMD-VSV-G and delta 8.2 plasmids were gifts from Dr. Dong (Tulane University, New Orleans, LA, USA). Red fluorescent protein (RFP) and *Mcl-1* cDNA lentiviral constructs were purchased from Dharmacon (Lafayette, CO, USA). Non-template negative control (NTC)-, *Bax*-, and *Bak*-shRNA lentiviral constructs were purchased from Sigma-Aldrich.

### Colony formation assay

Colony formation assays were carried out as previously described [32, 40]. Briefly, primary AML samples from patients and normal human CD34 + cord blood cells were treated with ONC213 for 48 h, then washed three times with PBS, plated in MethoCult (catalog number 04434; Stem Cell Technologies, Vancouver, Canada). Colony forming units (CFUs) were visualized 10–14 days later using an inverted microscope. Colonies containing more than 50 cells were counted. Technical triplicates were performed. Patient samples were chosen based on the availability of adequate sample for the assay.

### Statistical analysis

Unpaired t-test were used for comparisons between two different treatment groups. One-way ANOVA followed by Tukey post hoc test was used when comparing more than two groups. Overall survival was estimated using the Kaplan–Meier method, and statistical analysis was performed using the log-rank test. GraphPad Prism 9.0 was used to perform statistical analyses. Error bars represent mean ± SEM; significance level was set at *p* < 0.05.

## Results

### ONC213 + venetoclax has efficacy against AML cells in vitro and in vivo

**Fig. 1:**
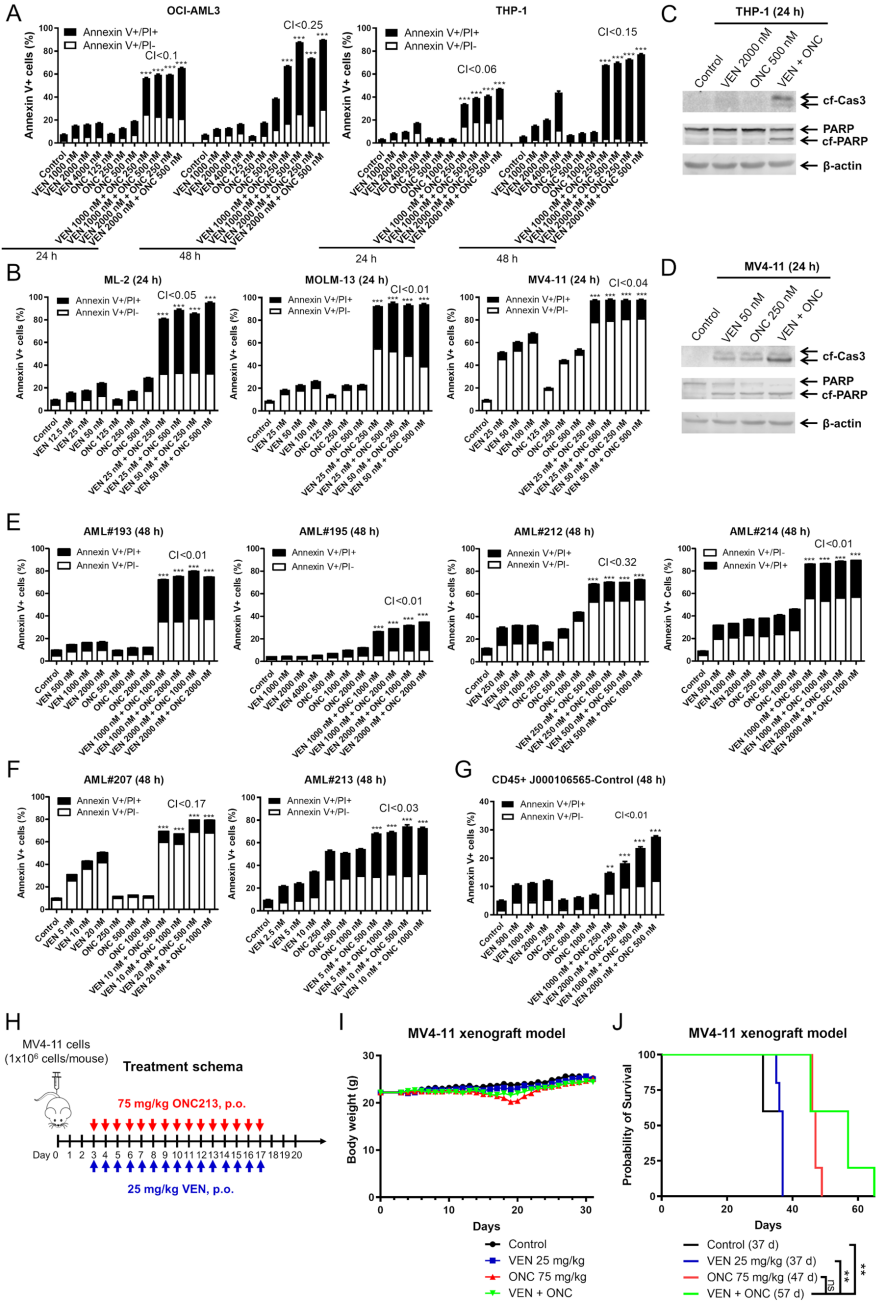
ONC213 + venetoclax has efficacy against AML cells in vitro and in vivo. (**A, B, E, F**) AML cells were treated with ONC213 (ONC) and venetoclax (VEN) for 24–48 h. The cells were then stained with annexin V/propidium iodide (PI) and analyzed via flow cytometry. ***indicates *p* < 0.001 compared to control and single drug treatments. Combination Index (CI) values were calculated using CompuSyn software. CI < 1.0, CI = 1.0, and CI > 1.0 indicate synergistic, additive, and antagonistic effects, respectively. (**C**&**D**) Representative western blots of whole cell lysates probed with the indicated antibodies are shown. cf-Cas3, cleaved form caspase 3; cf-PARP, cleaved form PARP. (**G**) J000106565 PDX cells freshly collected from NSGS mouse spleen were treated with ONC and VEN for 48 h and then stained with anti-human CD45 antibody, annexin V, and PI and analyzed by flow cytometry. Annexin V positive human CD45 + cells are graphed. ** *p* < 0.01 and *** *p* < 0.001 compared to control and single drug treatments. (**H-J**) MV4-11 cells were injected into NSGS mice via tail vein. On day 3, the mice were randomized into treatment arms (*n* = 5) and treated as indicated in panel H. Mouse body weights are graphed in panel I. The overall survival probability was estimated using the Kaplan-Meier method (panel J). ** *p* < 0.01

Treatment with up to 4000 nM of venetoclax for 24 h resulted in < 20% cell death in OCI-AML3 and THP-1 cells which are intrinsically resistant to venetoclax (Fig. 1A). When ONC213 (250 nM and 500 nM) was combined with venetoclax (1000 nM and 2000 nM) in these inherently resistant cell lines, there was significant and synergistic induction of cell death at 24 and 48 h (Fig. 1A). Synergy between the two agents were also detected in venetoclax-sensitive AML cell lines (ML-2, MOLM-13, and MV4-11), though the required concentrations of venetoclax were much lower (25 nM and 50 nM; Fig. 1B). Cleavage of PARP and caspase 3 were strongly enhanced in the combination when compared to ONC213 or venetoclax alone in THP-1 and MV4-11 cells, confirming enhanced induction of apoptosis in these cells (Fig. 1C and D).

To further assess the efficacy of ONC213 + venetoclax, we treated cells from newly diagnosed AML patients with ONC213 and venetoclax. The combination synergistically induced cell death in both venetoclax-resistant (500–4000 nM venetoclax treatment for 48 h did not result in significant cell death, Fig. 1E) and -sensitive primary AML patient samples (Fig. 1F). The combination also synergistically induced death of AML PDX cells (J000106565) treated ex vivo (Fig. 1G). These results support ONC213 + venetoclax for the treatment of AML cells regardless of venetoclax sensitivity.

Next, we determined the in vivo efficacy of ONC213 + venetoclax using a MV4-11 cell line derived xenograft mouse model. Dosage of ONC213 and venetoclax were determined from previous investigations [27, 39, 41] and through combination toxicity trials. NSGS mice were injected with MV4-11 cells via tail vein injection. Three days later the mice were randomized and treated with ONC213 (75 mg/kg, p.o.), venetoclax (25 mg/kg, p.o.), ONC213 + venetoclax, or vehicle control daily for 14 days (Fig. 1H). Body weights indicate that the combination treatment was well tolerated (Fig. 1I). The combination prolonged survival compared to venetoclax alone and vehicle control (57 vs. 37 days; *p* = 0.0034, Fig. 1J). Although the combined treatment resulted in 10 days longer survival (57 days vs. 47 days) compared to ONC213 alone, the difference was not significant (Fig. 1J). An additional study was conducted to test a different dosing and schedule; ONC213 was given at a higher dose less frequently (180 mg/kg, Q5D) and followed with venetoclax for 3 days (25 mg/kg, Figure S1A). This schedule was also well tolerated by the mice as evidenced by maintenance of body weight (Figure S1B), and the combination significantly enhanced survival of the mice compared to vehicle control and venetoclax alone (56.5 days vs. 41 days; *p* < 0.01) and ONC213 alone treated mice (56.5 days vs. 49 days; *p* = 0.0413) (Fig. S1C). Interestingly, in vitro treatment with venetoclax induced significant apoptosis (Fig. 1B), while venetoclax alone showed no survival benefit in vivo (Fig. 1J and Figure S1). This is likely due to many factors, including but not limited to the tumor microenvironment and pharmacokinetic properties. These studies establish the antileukemic efficacy of ONC213 and venetoclax in vitro and in vivo.

### ONC213 restores venetoclax sensitivity of AML cells

**Fig. 2:**
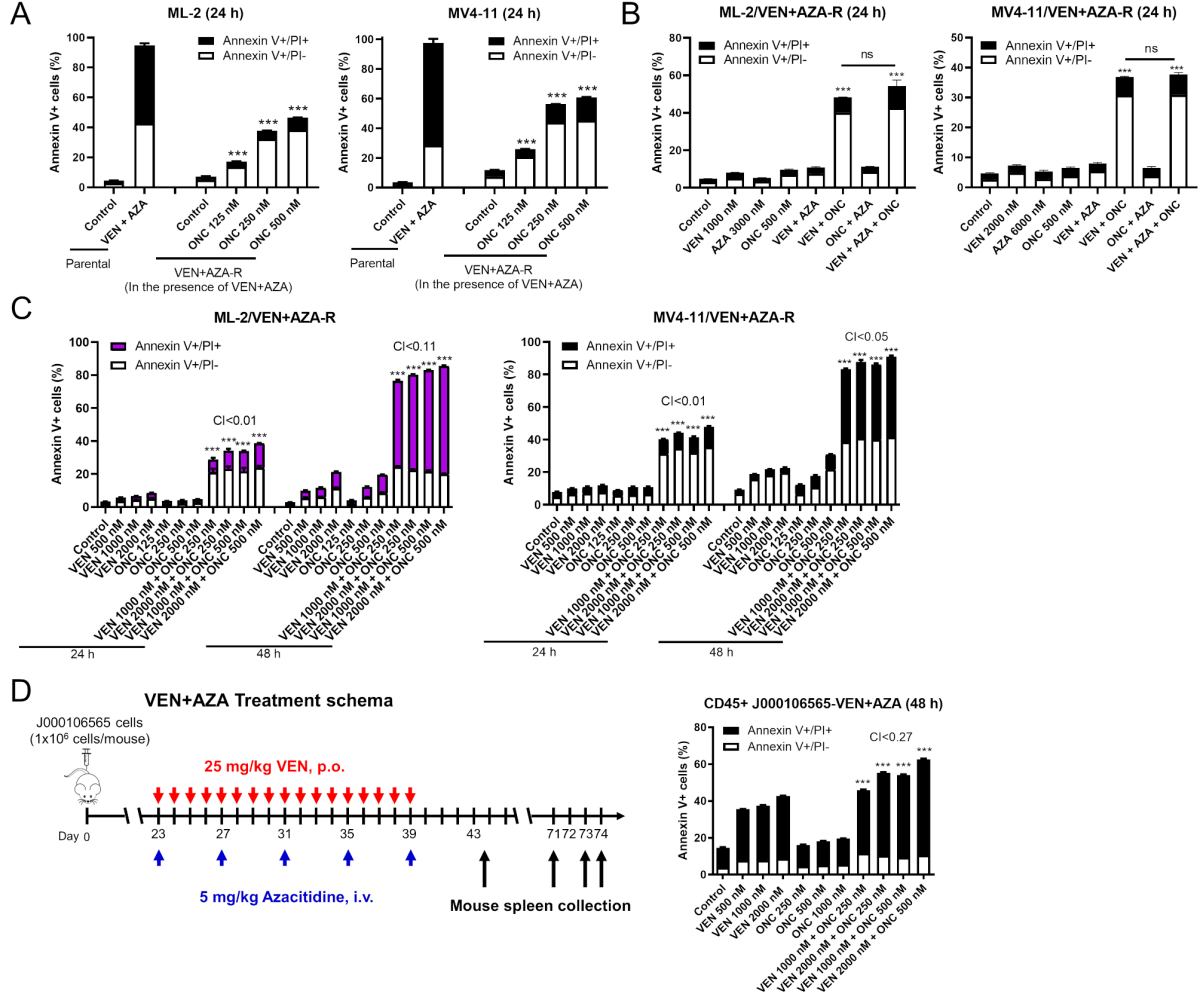
ONC213 resensitizes venetoclax-resistant cells to venetoclax. (**A**) ML-2/VEN + AZA-R and MV4-11/VEN + AZA-R cells cultured in the presence of venetoclax + azacitidine (VEN + AZA) were treated with ONC213 (ONC) for 24 h. The parental cells were also treated with VEN + AZA at concentrations that the resistant cells were cultured in for 24 h. The treated cells were stained with annexin V/PI and analyzed via flow cytometry. *** *p* < 0.001 compared to control. (**B**) ML-2/VEN + AZA-R and MV4-11/VEN + AZA-R cells cultured without VEN + AZA for 3 days were treated with VEN, AZA, ONC, or in combination for 24 h. The treated cells were stained with annexin V/PI and analyzed via flow cytometry. *** *p* < 0.001 compared to control and single drug treatments. ns indicates not significant, comparing VEN + ONC and VEN + AZA + ONC. (**C**) ML-2/VEN + AZA-R and MV4-11/VEN + AZA-R cells (without VEN + AZA present in the media for 72 h) were treated with VEN, ONC, or combination for 24 and 48 h, and then stained with annexin V/PI and analyzed via flow cytometry. Combination Index (CI) values were calculated using CompuSyn software to determine synergy. CI < 1.0, CI = 1.0, and CI > 1.0 indicate synergistic, additive, and antagonistic effects, respectively. *** *p* < 0.001 compared to vehicle control and single drug treatments. (**D**) J000106565 cells were injected intravenously through the tail vein of immunocompromised NSGS mice to generate a patient-derived xenograft model. Human cell engraftment was verified in three randomly selected mice 22 days later by flow cytometry for human CD45+ (hCD45+) cells in their peripheral blood (average 20.23%). On day 23, the mice were randomized (*n* = 5 mice/group) and treated as shown in the upper panel. Mice were sacrificed based on severity of disease-induced symptoms. Cells were isolated from spleens (VEN + AZA treated mice), treated and then stained with anti-hCD45 antibody, annexin V, and PI and analyzed via flow cytometry. Annexin V positive cells are graphed. ** *p* < 0.01 and *** *p* < 0.001 compared to control and single drug treatments. CI values were calculated as described in the description for panel C

To further investigate the potential of ONC213 + venetoclax, we determined the sensitivity of AML cell lines with acquired venetoclax + azacitidine-resistance [33]. Treatment with concentrations of venetoclax + azacitidine that the resistant lines are cultured in, induced 90% cell death in the parental cells but less than 10% cell death in the resistant lines. Addition of ONC213 to the culture media already containing venetoclax + azacitidine resulted in concentration-dependent induction of cell death (Fig. 2A). MOLM-13/VEN + AZA-R cells were developed by exposing MOLM-13 cells to increasing concentrations of VEN + AZA at a 1:3 ratio. The resulting cells have a VEN + AZA IC_50_ of 2734 nM (presented as the VEN concentration) with both drugs still in the culture media just prior to the MTT assay, and 2449 nM when both drugs were removed from culture for 3 days prior to MTT analysis (Figure S2A&B). These cells displayed increased expression of Mcl-1 and decreased expression of Bcl-2 and Bax compared to parental MOLM-13 cells (Figure S2C). Similar to the ML2/VEN + AZA-R and MV4-11/VEN + AZA-R cells, treatment with VEN + AZA (at the concentrations that the resistant line is maintained in) resulted in 90% cell death in the parental line and less than 10% in the MOLM-13/VEN + AZA-R cells (Figure S2D). Addition of ONC213 to the culture media already containing venetoclax + azacitidine resulted in concentration-dependent induction of cell death. Next, the ML2/VEN + AZA-R and MV4-11/VEN + AZA-R cells cultured in drug-free media for 3 days were treated with each drug alone and in all combinations. There was no significant induction of apoptosis following any single drug treatment or with the combination of venetoclax + azacitidine or ONC213 + azacitidine. There was significant apoptosis induction in both resistant lines following treatment with venetoclax + ONC213. However, this sensitivity was not further enhanced when azacitidine was added (Fig. 2B). These results suggest that in cells with acquired venetoclax + azacitidine-resistance, ONC213 resensitizes the cells to venetoclax. This resensitization was synergistic, as determined by flow cytometry analyses and the CompuSyn software (Fig. 2C). MOLM-13 and MV4-11 VEN resistant cell lines (designated MOLM-13/VEN-R and MV4-11/VEN-R, respectively) were generated by culturing the parental lines in media containing increasing concentrations of VEN. The resulting cells have VEN IC_50_s of approximately 4000–5000 nM, more than 100X that of the parental lines (Figure S3A). These cells displayed increased expression of Mcl-1 and decreased expression of Bax compared to parental cells (Figure S3B). These cells were then cultured in drug-free media for 3 days prior to treatment with VEN, ONC, or in combination. There was no significant induction of apoptosis following any single drug treatment, however, the combination significantly induced apoptosis in synergistic manner (CI < 0.01, Figure S3).

To further investigate the use of ONC213 + venetoclax against cells with acquired resistance to venetoclax + azacitidine, we utilized a PDX model that was developed from a patient who had undergone induction chemotherapy (J000106565). After confirmation of engraftment, mice were treated with azacitidine (5 mg/kg, i.v., Q4D), venetoclax (25 mg/kg, p.o., daily), or the combination of azacitidine and venetoclax for 17 days (Fig. 2D). Following treatment, mice were monitored for progression of leukemia symptoms and mouse spleens were harvested at the time of euthanasia (Fig. 2D). Overall, the treatment was well tolerated, as monitored by body weight, and the venetoclax + azacitidine-treated mice survived significantly longer than the control-, venetoclax-, and azacitidine-treated counterparts (74 vs. 43.5, 45.5, or 60 days, respectively, Figure S4). Mouse spleen cell suspensions from venetoclax + azacitidine-treated mice were treated with venetoclax and ONC213 ex vivo. While both monotherapies induced little to moderate cell death, the combination significantly and synergistically induced cell death (Fig. 2D). Here we found that ONC213 sensitizes venetoclax + azacitidine-resistant cells to venetoclax, similar to inherently venetoclax-resistant AML cell lines (THP-1 and OCI-AML3).

### ONC213 + venetoclax target AML progenitor and stem cells

**Fig. 3:**
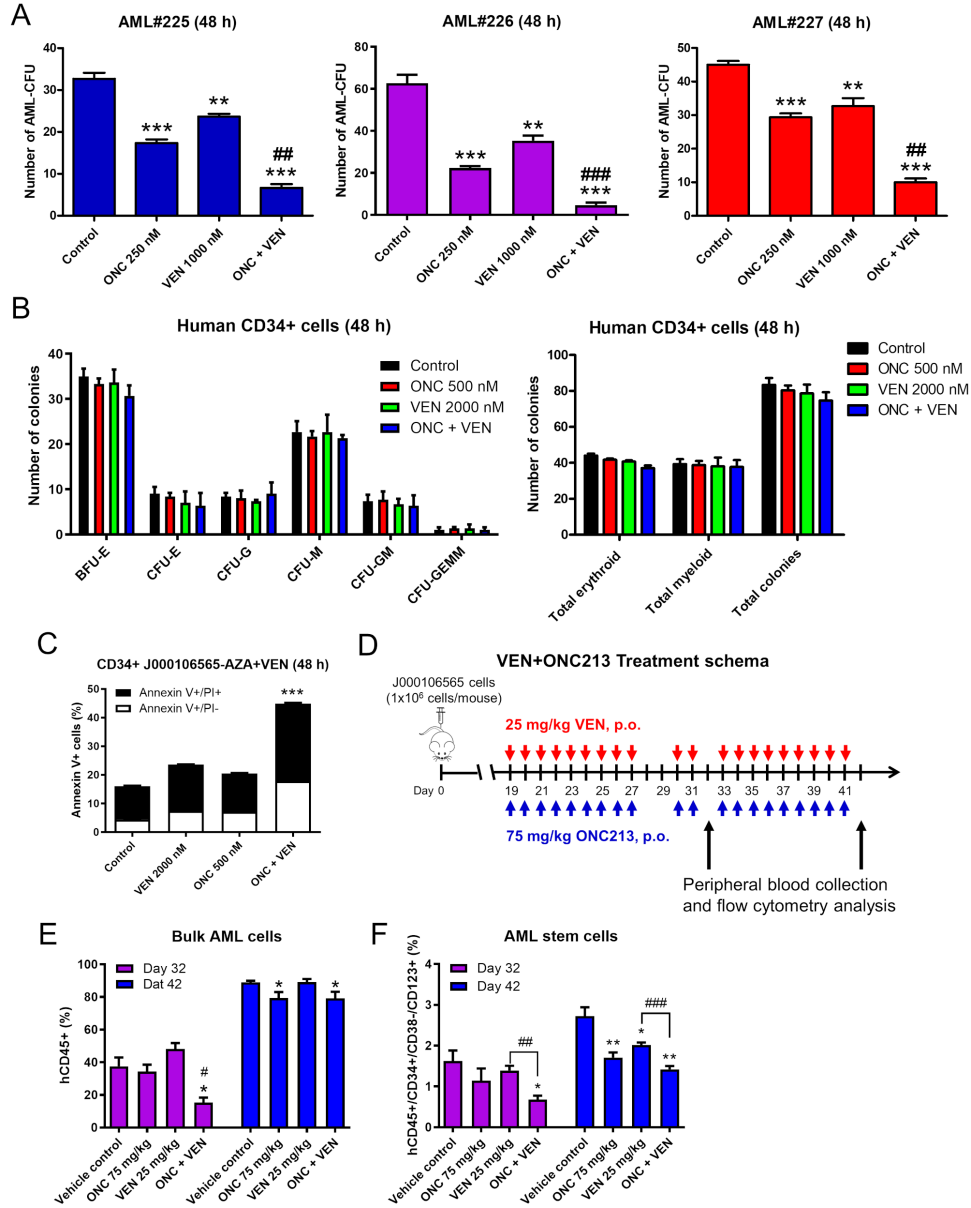
ONC213 and venetoclax target AML progenitor and stem cells. (**A**) Primary AML patient samples were cultured with ONC213 (ONC) and venetoclax (VEN), alone or in combination, for 48 h and then plated in methylcellulose and incubated for 2 weeks. The number of surviving AML cells capable of generating leukemia colonies (AML-CFUs) were enumerated. Technical triplicates were performed. Data are presented as mean ± SEM. ** *p* < 0.01 and *** *p* < 0.001 compared to vehicle control; ## *p* < 0.01 and ### *p* < 0.001 compared to single drug treatments. (**B**) Human CD34 + cord blood cells were treated with ONC213 and VEN, alone or in combination, for 24 h and then plated in methylcellulose and incubated for 2 weeks. The number of surviving normal hematopoietic cells capable of generating colonies were counted and colonies are presented as mean ± SEM. The number of BFU-E, CFU-E, CFU-G, CFU-M, CFU-GM, and CFU-GEMM is presented as mean ± SEM (left panel). Total erythroid and myeloid colonies are presented as mean ± SEM (right panel). (**C**) AML cells were isolated from spleens of NSGS mice injected with J000106565 patient-derived AML cells and treated with VEN + AZA. CD34 MicroBeads were used to enrich human CD34 + cells. These cells were treated with VEN, ONC, or the combination for 48 h, and then stained with anti-human CD45 and annexin V/PI and analyzed via flow cytometry. *** *p* < 0.001 compared to control and single drug treatments. (**D-F**) NSGS mice were injected with J000106565 patient-derived AML cells. Engraftment was confirmed on day 18. On day 19, mice were randomized into treatment groups (*n* = 5) and treated as shown in panel D. On days 32 (part way through treatment) and 42 (at the end of treatment) peripheral blood was collected, and cells were stained with anti-human CD45, -CD34, -CD38, and -CD123 antibodies and analyzed via flow cytometry. Bulk AML cells were determined by gating for CD45 + cells (panel E). AML stem cells were determined by gating for CD45dim+/CD34+/CD38-/CD123 + cells (panel F). * *p* < 0.05 and ** *p* < 0.01 compared to control. # *p* < 0.05 compared to single drug treatments (left panel). ## *p* < 0.01 and ### *p* < 0.001 compared to VEN treatment (right panel)

We have previously reported that ONC213 targets AML progenitor cells, so we sought to determine the effect of ONC213 + venetoclax on AML progenitor cells [27]. Primary patient AML cells isolated from bone marrow samples were treated with ONC213 (250 nM) and venetoclax (1000 nM), alone or in combination for 48 h. Colony formation assay results show that compared to untreated control cells, both venetoclax and ONC213, alone or combined, significantly reduced AML progenitor cells (Fig. 3A). Further, the combination further significantly reduced AML progenitors compared to the single treatments (Fig. 3A). The combination showed no significant effect on normal (non-malignant) human CD34 + cells even at higher concentrations of both ONC213 (500 nM) and venetoclax (2000 nM, Fig. 3B). To further elucidate the ability of the combination to target progenitor and stem cells, we collected spleens from the J000106565 PDX mice treated with venetoclax + azacitidine (Fig. 2D) and isolated CD34 + cells using magnetic beads to enrich the progenitor/stem cell population. Although both monotherapies showed minimal effect on CD34 + AML cells, ONC213 + venetoclax significantly induced cell death (Fig. 3C).

To test ONC213 + venetoclax on AML stem cells in vivo, NSGS mice were injected with J000106565 cells. Engraftment was confirmed (day 18) and then mice were randomized and treated with ONC213 (75 mg/kg, p.o.), venetoclax (25 mg/kg, p.o.), or the combination, daily for 22 days (with exception of a drug holiday on days 28–29 and on days in which blood was drawn, Fig. 3D). Peripheral blood was collected from the submandibular vein, during treatment (day 32) and immediately following cessation of treatment (day 42). Both the percentage of circulating bulk AML cells (hCD45+) and AML stem cells (hCD45+/hCD34+/hCD38-/hCD123+) were assessed. On day 32, the combination of ONC213 and venetoclax significantly reduced the number of circulating bulk AML cells (CD45+) compared to the control and monotherapy treated mice and significantly reduced circulating AML stem cells compared to control and venetoclax treated mice (Fig. 3E&F). By day 42 though, the effect of the combination therapy on bulk AML cells was blunted (following the last treatment) where control and venetoclax treated mice had nearly 90% hCD45 + cells and ONC213 + venetoclax treated mice had almost 80% (Fig. 3E). However, the effect of the combination on AML stem cells was still evident on day 42 (Fig. 3F). As expected with the similar percentage of circulating AML cells, there was no difference in survival between the treated groups (data not shown). These results suggest that ONC213 + venetoclax targets AML stem/progenitor cells, including AML cells with resistance to venetoclax + azacitidine.

### ONC213 induces a mitochondrial stress response and combination treatment diminishes mitochondria function in AML cells

**Fig. 4:**
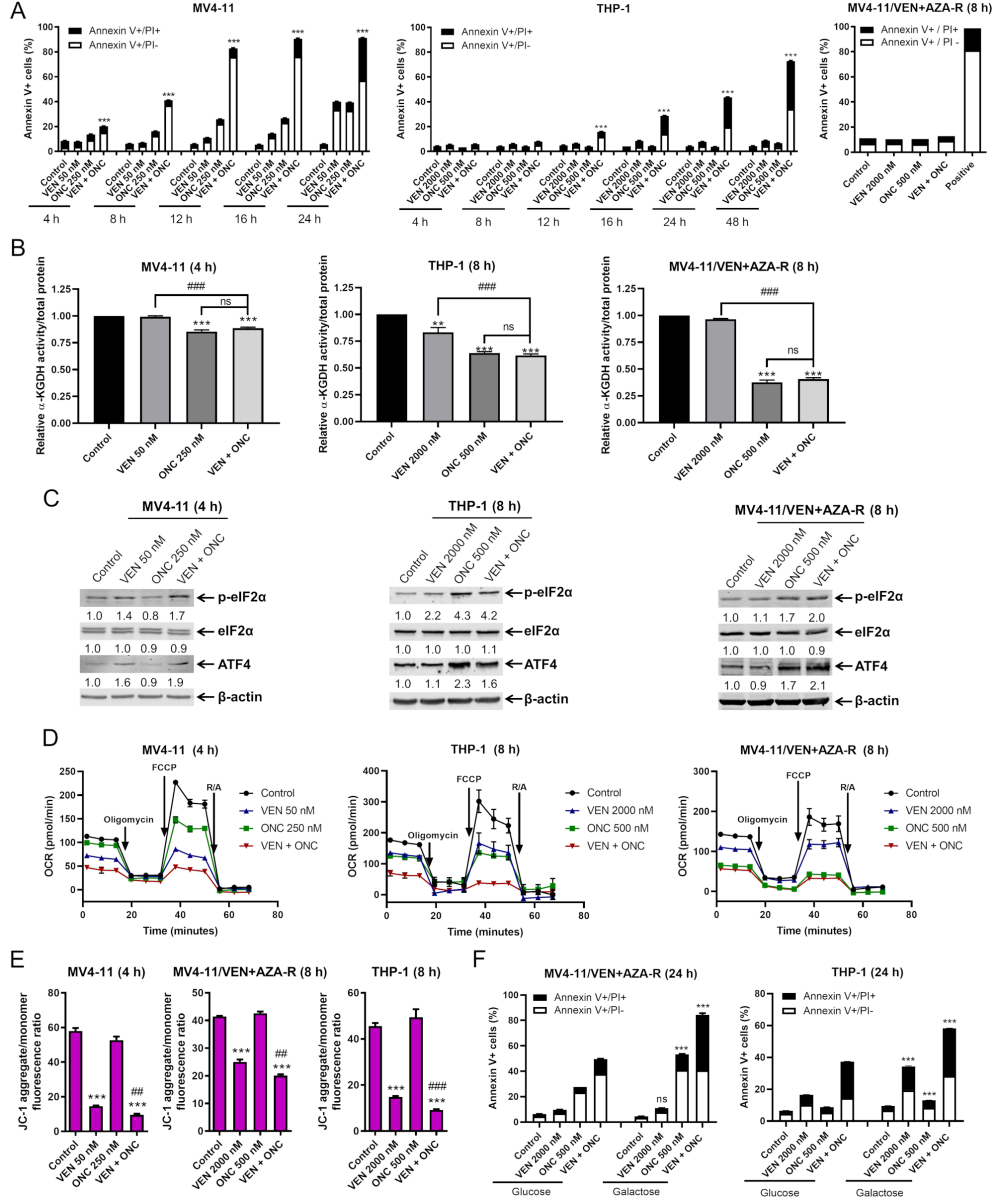
ONC213 induces a mitochondrial stress response and its combination with venetoclax diminishes mitochondria function in AML cells. (**A**) MV4-11, THP-1, and MV4-11/VEN + AZA-R cells were treated with venetoclax (VEN), ONC213 (ONC), or ONC + VEN for up to 48 h and then stained with annexin V/PI and analyzed via flow cytometry. *** indicates *p* < 0.001 compared to control and single drug treatments. (**B**) MV4-11, THP-1, MV4-11/VEN + AZA-R cells were treated with VEN and ONC, alone or in combination, for 4–8 h. α-ketoglutarate dehydrogenase (α-KGDH) activity was measured. The ratio of α-KGDH activity to total protein was determined and normalized to the vehicle control. ** *p* < 0.01 and *** *p* < 0.001 compared to control. ### *p* < 0.001 compared to VEN. ns not significant compared to ONC. (**C**) MV4-11, THP-1, and MV4-11/VEN + AZA-R cells were treated with VEN and ONC, alone or in combination, for 4–8 h. Representative western blots of whole cell lysates probed with the indicated antibodies are shown. The fold changes for the densitometry measurements, normalized to β-actin and then compared to vehicle control, are indicated below the corresponding blots. (**D**) MV4-11, THP-1, and MV4-11/VEN + AZA-R cells were treated with VEN and ONC, alone or in combination, for 4–8 h and then subjected to the cellular mito stress test. OCR was measured following injection with oligomycin A (complex V inhibitor), FCCP (mitochondrial membrane uncoupler), and rotenone + antimycin A (complex I and III inhibitors). (**E**) MV4-11, THP-1, and MV4-11/VEN + AZA-R cells were treated with VEN and ONC, alone or in combination, for 4–8 h and then stained with JC-1 and analyzed via flow cytometry for aggregate vs. monomer fluorescence ratio. *** *p* < 0.001 compared to control. ## *p* < 0.01 and ### *p* < 0.001 compared to ONC alone and VEN alone. (**F**) MV4-11/VEN + AZA-R and THP-1 cells were cultured in either glucose or galactose containing media for 48 h and then treated with VEN, ONC, or the combination of ONC + VEN for 24 h. Then the cells were stained with annexin V/PI and analyzed via flow cytometry for apoptosis. ns not significant and *** *p* < 0.001 compared to the same treatment in glucose media

To understand the mechanisms responsible for the synergy between ONC213 + venetoclax, we investigated the role of mitochondrial stress. To ensure we captured cellular changes prior to onset of cell death, we performed time course analysis (Fig. 4A). The following experiments were conducted prior to the onset of cell death; 4 h for MV4-11 and 8 h for THP-1 and MV4-11/VEN + AZA-R. We previously reported that ONC213 targets mitochondrial metabolism partially through suppression of alpha-ketoglutarate dehydrogenase (α-KDGH), an important enzyme in the citric acid cycle [27]. Thus, we evaluated the effect of ONC213, venetoclax, and ONC213 + venetoclax on α-KDGH activity prior to the onset of cell death. In MV4-11 and THP-1 cells, ONC213 and ONC213 + venetoclax significantly reduced α-KDGH activity compared to the control and venetoclax alone (Fig. 4B). The results are more striking in MV4-11/VEN + AZA-R cells where ONC213 reduced α-KDGH activity by over 50% (Fig. 4B). We also previously demonstrated that ONC213 induces a stress response in AML cells, including increasing phosphorylation of eIF2α and levels of ATF4 [27]. In MV4-11 (venetoclax-sensitive) and THP-1 (venetoclax-resistant), there was a substantial increase in phosphorylation of eIF2α (p-eIF2α) in ONC213 + venetoclax treated cells with insubstantial changes to eIF2α levels (Fig. 4C). Additionally, there is evidence of ATF4 induction in response to ONC213 + venetoclax (Fig. 4C). Similar results were obtained in MV4-11/VEN + AZA-R cells (Fig. 4C). This demonstrates that the induction of mitochondrial stress is maintained in the combination therapy and is present in cells that are resistant to venetoclax + azacitidine.

Next, we investigated the effect of ONC213 + venetoclax on mitochondrial respiration using a Seahorse bioanalyzer. In MV4-11 cells, venetoclax (50 nM) and ONC213 (250 nM) significantly reduced basal respiration, maximal respiration, and spare respiratory capacity compared to untreated control cells after 4 h of treatment (Fig. 4D and S5A). ONC213 + venetoclax further significantly reduced all three parameters compared to either treatment alone (*p* < 0.001). Similar results were achieved in both THP-1 and MV4-11/VEN + AZA-R cells after 8 h of treatment (Fig. 4D and S5B&C). This was accompanied by significantly reduced mitochondrial membrane potential determined by JC-1 staining and flow cytometry analysis (Fig. 4E).

To elucidate the role of mitochondrial respiration in cell death induced by ONC213 + venetoclax, we cultured MV4-11/VEN + AZA-R and THP-1 in normal glucose containing media and in media without glucose but supplemented with galactose to force oxidative phosphorylation reliance [42]. In MV4-11/VEN/AZA-R cells, there was a significant increase in apoptosis in cells cultured in galactose media when treated both with ONC213 and ONC213 + venetoclax. Similar results were obtained in THP-1 cells, with an additional increase in apoptosis in cells treated with venetoclax alone, as well (Fig. 4F).

### ONC213 + venetoclax induced cell death is partially dependent on Mcl-1

**Fig. 5:**
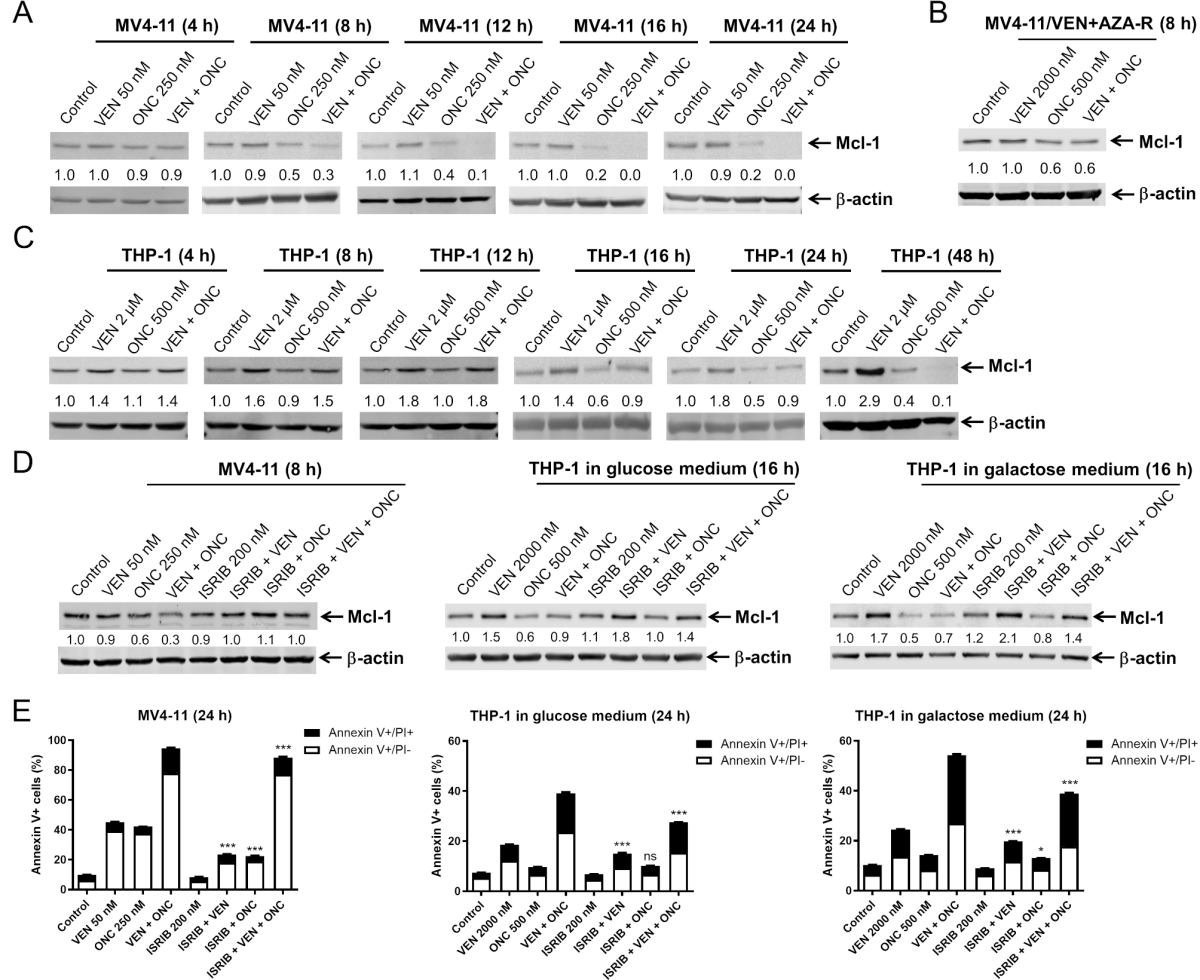
ONC213 + venetoclax induced cell death is partially dependent on Mcl-1. (**A-C**) MV4-11, THP-1, and MV4-11/VEN + AZA-R cells were treated with VEN and ONC, alone or in combination, for up to 48 h. Representative western blots of whole cell lysates probed with the indicated antibodies are shown. The fold changes for the densitometry measurements, normalized to β-actin and then compared to vehicle control, are indicated below the corresponding blots. (**D**) MV4-11 cells and THP-1 cells (cultured in glucose or galactose containing media) were treated with VEN, ONC, or ISRIB, alone or in combination, for 8–16 h. Representative western blots of whole cell lysates probed with the indicated antibodies are shown. The fold changes for the densitometry measurements, normalized to β-actin and then compared to vehicle control, are indicated below the corresponding blots. (**E**) MV4-11 cells and THP-1 cells (cultured in glucose or galactose containing media) were treated with VEN, ONC, or ISRIB, alone or in combination, for 24 h and then stained with annexin V/PI and analyzed via flow cytometry. * *p* < 0.05, *** *p* < 0.001, and ns not significant compared to the same drug treatment but without ISRIB

We have previously determined that ONC213 treatment leads to Mcl-1 reduction [27], so we sought to determine its’ role in ONC213’s synergy with venetoclax. We found that in MV4-11 cells, ONC213 both alone and in combination with venetoclax, reduced Mcl-1 levels as early as 8 h post-treatment and this reduction is maintained throughout 24 h (Fig. 5A). Similar to MV4-11, treatment of MV4-11/VEN + AZA-R cells with ONC213 alone or in combination with venetoclax for 8 h resulted in decreased Mcl-1 levels (Fig. 5B). In THP-1 cells, Mcl-1 is decreased under ONC213 alone as early as 16 h post-treatment (Fig. 5C). As previously reported [39, 43], venetoclax induced Mcl-1 in THP-1 cells which was completely abolished 16 h post the addition of ONC213. Importantly, all reductions in Mcl-1 in these cell lines occur at the same time or prior to significant induction of apoptosis (Fig. 4A). Here we find that ONC213 maintains induction of stress response proteins in the presence of venetoclax and this is associated with reduction of Mcl-1 protein, a known cause of venetoclax resistance.

To investigate if reduction of Mcl-1 protein levels could be rescued through inhibition of the stress response pathway, we utilized integrated stress response inhibitor (ISRIB), which is a small molecule inhibitor that works through stabilization of eIF2B, allowing protein synthesis to continue even under stress conditions [44–48]. As expected, Mcl-1 levels were substantially reduced with ONC213 and ONC213 + venetoclax treatment which was completely abolished by the addition of ISRIB in MV4-11 (Fig. 5D). Cells with resistance to venetoclax + azacitidine have been found to have altered metabolic profiles, including an increased utilization of glycolytic pathways [33, 49–51]. Additionally, Mcl-1 has been revealed to have many roles in addition to inhibition of the intrinsic apoptosis pathway, including regulating cellular metabolism and oxidative phosphorylation [52]. To further investigate the role of metabolism in the context of Mcl-1, we incubated THP-1 cells in either normal media, containing glucose, or in media containing only galactose as a sugar source. ISRIB almost completely canceled the downregulation of Mcl-1 by ONC213 and ONC213 abolishment of Mcl-1 induction by venetoclax in THP-1 cells cultured in media supplemented with glucose or galactose (Fig. 5D). In MV4-11 cells cultured in glucose media and THP-1 cells cultured in glucose or galactose media, addition of ISRIB significantly rescued cells from venetoclax, ONC213, and ONC213 + venetoclax induced cell death (Fig. 5E). Notably though, the rescue effect by ISRIB in MV4-11 cells treated with the combination was minimal and still resulted in cell death greater than 88%. It is also notable that there was an overall increase in induction of THP-1 cell death compared to cells incubated in glucose media.

### Induction of mitochondrial stress via ONC213 supports intrinsic apoptosis activation via venetoclax

**Fig. 6:**
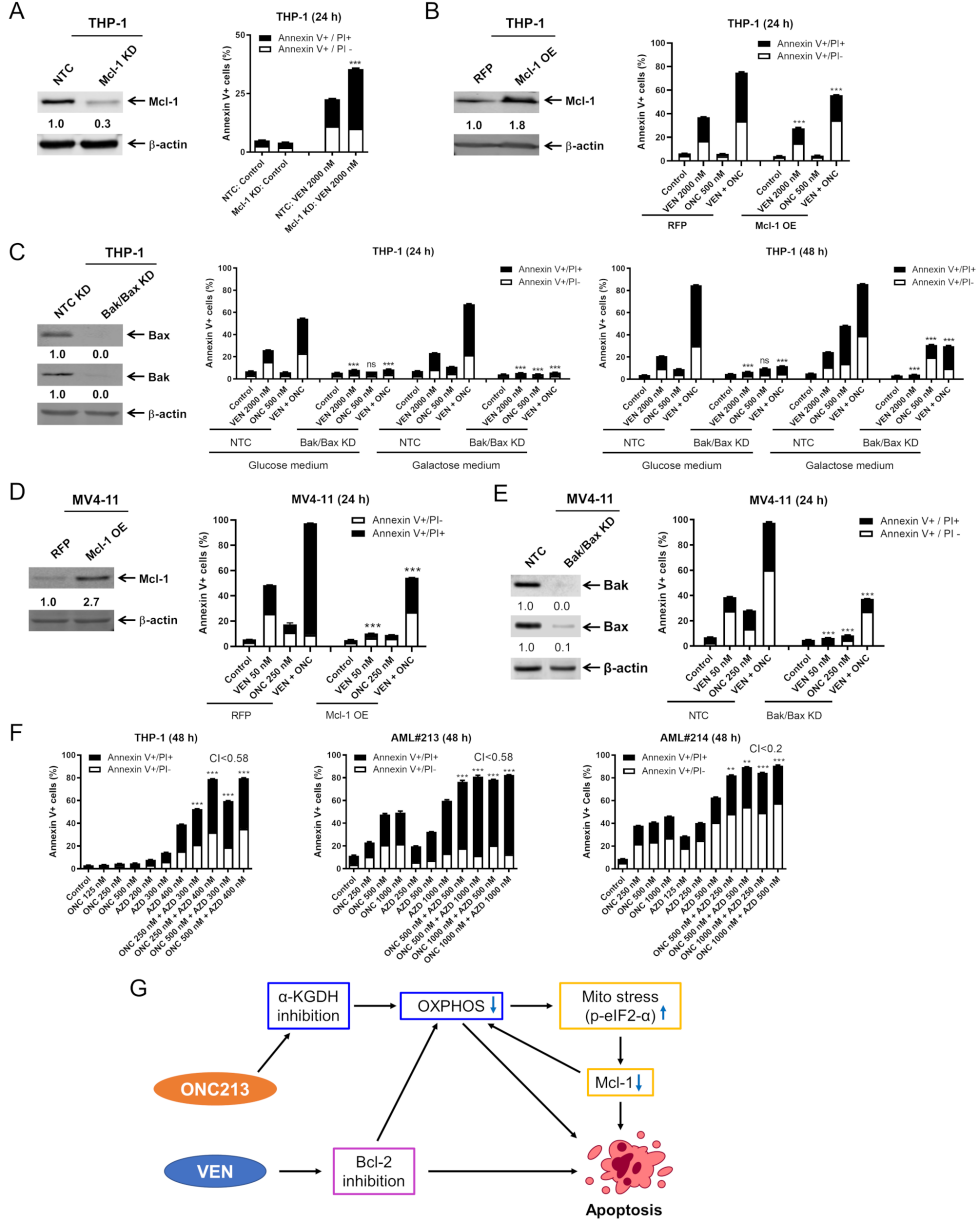
Induction of mitochondrial stress via ONC213 supports intrinsic apoptosis activation via venetoclax. (**A**) CRISPR knockdown (KD) of Mcl-1 and non-target control (NTC) was generated using THP-1 cells. Representative western blots of whole cell lysates probed with the indicated antibodies are shown in the left panel. The fold changes for the Mcl-1 densitometry measurements, normalized to β-actin and then compared to NTC, are indicated. Cells were treated with vehicle control or venetoclax (VEN) for 24 h. Flow cytometry results of annexin V/PI staining are show in the right panel. *** *p* < 0.001 compared to NTC treated with VEN. (**B**) THP-1 RFP (red fluorescent protein) and Mcl-1 OE (overexpression) whole cell lysates were subjected to western blot analysis. The fold changes for the Mcl-1 densitometry measurements, normalized to β-actin and then compared to RFP, are indicated. Cells were treated with vehicle control, VEN, ONC, or in combination for 24 h. Flow cytometry results of annexin V/PI staining are show in the right panel. *** *p* < 0.001 compared to RFP cells under the same treatment conditions. (**C**) THP-1 NTC and Bak/Bax KD whole cell lysates were subjected to western blot analysis. The fold changes for the Bax and Bak densitometry measurements, normalized to β-actin and then compared to NTC, are indicated. THP-1 NTC and Bak/Bax KD cells were cultured in either glucose or galactose containing media for 48 h and then treated with VEN, ONC213, or the combination of ONC + VEN for 24–48 h. Then the cells were stained with annexin V/PI and analyzed via flow cytometry. *** *p* < 0.001 and ns not significant compared to NTC cells under the same treatment conditions. (**D**) MV4-11 RFP and Mcl-1 OE whole cell lysates were subjected to western blot analysis. The fold changes for the Mcl-1 densitometry measurements, normalized to β-actin and then compared to RFP, are indicated. Cells were treated with vehicle control, VEN, ONC, or in combination for 24 h. Flow cytometry results of annexin V/PI staining are show in the right panel. *** *p* < 0.001 compared to RFP cells under the same treatment conditions. (**E**) MV4-11 NTC and Bak/Bax KD whole cell lysates were subjected to western blot analysis. The fold changes for the Bax and Bak densitometry measurements, normalized to β-actin and then compared to NTC, are indicated. MV4-11 NTC and Bak/Bax KD cells were cultured in glucose containing media for 48 h and then treated with VEN, ONC, or the combination of ONC + VEN for 24 h. Then the cells were stained with annexin V/PI and analyzed via flow cytometry. *** *p* < 0.001 compared to NTC cells under the same treatment conditions. (**F**) THP-1 cells and primary AML patient samples AML#213 and AML#214 were treated with VEN, ONC, or VEN + ONC for 48 h. The cells were then stained with annexin V/PI and subjected to flow cytometry analyses. Combination Index (CI) values were calculated using CompuSyn software to determine synergy. CI < 1.0, CI = 1.0, and CI > 1.0 indicate synergistic, additive, and antagonistic effects, respectively. ** *p* < 0.01 and *** *p* < 0.001 compared to control and single drug treatments. (**G**) Proposed mechanism of action of ONC + VEN. ONC213 targets oxidative phosphorylation via inhibition of α-KGDH which subsequently induces mitochondrial stress pathways (p-eIF2-α) and reduces levels of Mcl-1. Mcl-1 is a known contributor to resistance to venetoclax and reduction of Mcl-1 activates the intrinsic apoptosis pathway and contributes to reduction of oxidative phosphorylation, enhancing the antileukemic activity of venetoclax against AML

To better understand the role that Mcl-1 plays in response to ONC213 + venetoclax, we knocked down Mcl-1 in THP-1 cells (Fig. 6A). Knockdown of Mcl-1 significantly sensitized THP-1 cells to venetoclax treatment (2000 nM, 24 h) in comparison to NTC control cells. To see if increasing the expression of Mcl-1 could protect cells from induction of apoptosis by combination therapy, we overexpressed Mcl-1 in THP-1 cells (Fig. 6B). This resulted in a significant reduction in apoptosis in THP-1 Mcl-1 OE (overexpression) cells in response to ONC213 + venetoclax treatment in comparison to the THP-1 RFP control cells (Fig. 6B). There was also a significant reduction in apoptosis in THP-1 Mcl-1 OE cells compared to RFP control cells following venetoclax treatment (Fig. 6B).

Given these results and increased response to the combination when glycolysis is deterred, we investigated the importance of both the intrinsic apoptosis pathway and cellular metabolism in response to the combination of ONC213 + venetoclax. First, we knocked down Bak and Bax, two pro-apoptotic proteins that are essential for induction of the intrinsic apoptosis pathway, in THP-1 cells (Fig. 6C). Under both glucose and galactose media conditions, we treated THP-1 NTC control and Bak/Bax KD cells with ONC213, venetoclax, or ONC213 + venetoclax for 24 and 48 h. In glucose supplemented media, knockdown of Bak and Bax significantly blunted induction of apoptosis following treatment with ONC213 + venetoclax compared to NTC control cells at both 24 and 48 h, with virtually no induction of apoptosis compared to untreated control cells (Fig. 6C). The knockdown of Bak and Bax similarly significantly reduced induction of apoptosis in response to the combination of ONC213 + venetoclax when cells were cultured in galactose media at both 24 and 48 h. However, there was significant induction of apoptosis in both NTC and Bak/Bax KD cells cultured in galactose treated with venetoclax, ONC213, and ONC213 + venetoclax at 48 h compared to cells cultured with glucose (Fig. 6C). Specifically, in cells treated with ONC213 + venetoclax for 48 h, there was about a 20% increase in cell death in galactose supplemented Bak/Bax KD cells compared to Bak/Bax KD cells cultured in glucose media.

We repeated these experiments in MV4-11 cells to examine if this effect was reproducible in cells that are both inherently sensitive to venetoclax therapy and less glycolytic than THP-1 cells. Compared to RFP control cells, Mcl-1 overexpression in MV4-11 cells significantly reduced induction of apoptosis in response to both venetoclax and ONC213 + venetoclax (Fig. 6D). To inhibit induction of the intrinsic apoptosis pathway, both Bak and Bax were knocked down in MV4-11 cells (Fig. 6E). MV4-11 NTC and Bak/Bax KD cells were incubated with ONC213, venetoclax, or ONC213 + venetoclax for 24 h. There was significant reduction in apoptosis in all treated Bak/Bax KD cells compared to NTC control cells. However, Bak and Bax double KD did not completely abolish apoptosis induction by ONC213 + venetoclax. To further confirm the role of Mcl-1 in ONC213 + venetoclax induced cell death, THP-1 cells and two primary AML patient samples were treated with ONC213 in combination with the Mcl-1 inhibitor AZD5991. The combination treatment resulted in synergistic induction of cell death (Fig. 6F). These results demonstrate that activation of the intrinsic apoptosis pathway via targeting Mcl-1 is important for cell death induced by ONC213 + venetoclax. The data also show that when cells are more reliant on oxidative phosphorylation for energy, as is the case with MV4-11 cells, and when cells have glycolytic pathways limited, they have increased sensitivity to ONC213 + venetoclax.

## Discussion

In our previous study, we demonstrated that ONC213 suppresses α-KGDH which induces a potent ISR, causing suppression of protein translation which results in decrease of Mcl-1, an important protein in resistance to venetoclax [27]. Here, we demonstrate that this downregulation of Mcl-1 plays an important role in the synergistic antileukemic activity of ONC213 + venetoclax. Our results are consistent with Sharon et al. who reported that inhibition of mitochondrial translation enhances venetoclax activity against AML cells [53]. Our results are also similar to those by Nii et al. who found that combination of the imipridone ONC212 with venetoclax induces AML cell apoptosis and prolonged survival in a cell line-derived xenograft model [54]. While both ONC212 and ONC213 are imipridones with similar structures and both induce ISR, their mechanisms of action have some differences including activation of CHOP which occurs in ONC212 treated but not ONC213 treated AML cells [27, 54].

LSCs are difficult to target therapeutically, but are associated with disease recurrence, resistance to therapy, and poor clinical prognosis [22, 24]. However, LSCs favor oxidative phosphorylation over glycolysis and have impaired glycolytic capacity compared to normal hematopoietic stem cells [17–19, 55–57]. While the combination of venetoclax and azacitidine has been shown to reduce oxidative phosphorylation in AML LSCs through inhibition of amino acid uptake [18, 56], the majority of patients who initially respond to this combination relapse within 18 months and many patients will fail to achieve complete remission [10]. Following treatment with venetoclax and azacitidine, LSCs can upregulate fatty acid metabolism to overcome amino acid uptake inhibition, resulting in unhindered oxidative phosphorylation and LSC survival [51]. Here we show evidence that the combination of ONC213 and venetoclax has antileukemic activity against LSCs (Fig. 3F). Furthermore, ex vivo treatment of AML cells resistant to venetoclax + azacitidine with ONC213 + venetoclax resulted in synergistic induction of AML cell death (Fig. 2D). This demonstrates that ONC213 and venetoclax targets AML progenitor/stem cells, which are known barriers to long term survival in AML patients and a significant cause of disease relapse and recurrence. However, the limited activity against bulk AML cells indicates that this combination should be tested with or after an effective debulking therapy.

Our investigations here also demonstrate that when cells are more reliant on oxidative phosphorylation, as is the case with LSCs, there is an increased sensitivity to ONC213 + venetoclax that cannot be overcome through halting ISR. Importantly, even when ISR pathway is blunted via ISRIB, there is still significant induction of apoptosis in all cell lines treated with ONC213 + venetoclax which demonstrates a multifactorial mechanism (Fig. 5E). Given the upregulation of Mcl-1 and increased reliance VEN + AZA resistant cells have on glycolysis, dysregulation of glycolysis induced by mitochondrial stress following ONC213 + venetoclax in AML cells is a likely contributing factor to initiation of cell death. This is especially compelling given that previous studies have indicated that Mcl-1 contributes to changes in metabolism and metabolic adaptations are essential to development of resistance [49].

## Conclusions

In summary, here we demonstrate that ONC213 can resensitize VEN + AZA-resistant AML cells to venetoclax therapy and target LSCs ex vivo and in vivo. ONC213 asserts its effectiveness on AML cells through targeting oxidative phosphorylation via inhibition of α-KGDH which subsequently induces mitochondrial stress pathways (p-eIF2-α) and reduces levels of Mcl-1. Mcl-1 is a known contributor to resistance to venetoclax and reduction of Mcl-1 supports promotion of the intrinsic apoptosis pathway and contributes to reduction of oxidative phosphorylation. ONC213 and venetoclax act synergistically to target AML cells and offer a unique and promising approach to combating the clinical challenge of resistance to venetoclax and HMA therapies (Fig. 6G).

## Electronic supplementary material

Below is the link to the electronic supplementary material.

**Additional File 1**: Supplementary Methods and Data. Supplementary methods. Figures S1-S5, and Table S1.

## Data Availability

All data reported in this paper are available within the article and its supplement or will be provided upon request to Yubin Ge (gey@karmanos.org).

## Abbreviations

AML: Acute myeloid leukemia
LSCs: Leukemia stem cells
PDX: Patient-derived xenograft
OS: Overall survival
LDAC: Low-dose cytarabine
HMAs: Hypomethylating agents
IC: Intensive chemotherapy
ISR: Integrated stress response
FITC: Fluorescein isothiocyanate
PI: Propidium iodide
CI: Combination index
cf: Cleaved
CMST: Cellular mito stress test
NTC: Non-template negative control
CFUs: Colony forming units
α-KDGH: Alpha-ketoglutarate dehydrogenase
ISRIB: Integrated stress response inhibitor
RFP: Red fluorescent protein
OE: Overexpression
