# Supplementary material for: ONC213: a novel strategy to resensitize resistant AML cells to venetoclax through induction of mitochondrial stress
Source: J Exp Clin Cancer Res. 2025 Jan 9;44:10. doi: 10.1186/s13046-024-03267-6 (PMC11714820; doi:10.1186/s13046-024-03267-6)
Supplement: Supplementary file 1 — Additional File 1: Supplementary Methods and Data. Supplementary methods. Figures S1-S5, and Table S1. [file 13046_2024_3267_MOESM1_ESM.pdf]

ONC213: A Novel Strategy to Resensitize Resistant AML Cells to Venetoclax Through Induction of Mitochondrial Stress

Jenna L. Carter[1,2], Yongwei Su[3,4], Eman T. Al-Antary[5,6], Jianlei Zhao[3,4], Xinan Qiao[7], Guan Wang[7], Holly Edwards[3,4], Lisa Polin[3,4], Juiwanna Kushner[3,4], Sijana H Dzinic[3,4], Kathryn White[3,4], Steven A Buck[8], Maik Hüttemann[1,9], Joshua E. Allen[10], Varun V. Prabhu[10], Jay Yang[3,4], Jeffrey W. Taub[4,5,6,8,*] and Yubin Ge[1,3,4,*]

[1]Cancer Biology Graduate Program, Wayne State University School of Medicine, Detroit, MI 48201, USA
[2]MD/PhD Program, Wayne State University School of Medicine, Detroit, MI 48201, USA
[3]Department of Oncology, Wayne State University School of Medicine, Detroit, MI 48201, USA
[4]Molecular Therapeutics Program, Barbara Ann Karmanos Cancer Institute, Wayne State University School of Medicine, Detroit, MI 48201, USA
[5]Division of Pediatric Hematology/Oncology, Children's Hospital of Michigan, Detroit, MI 48201, USA
[6]Department of Pediatrics, Central Michigan University College of Medicine, Mt. Pleasant, MI 48859, USA
[7]National Engineering Laboratory for AIDS Vaccine, School of Life Sciences, Jilin University, Changchun, China
[8]Department of Pediatrics, Wayne State University School of Medicine, Detroit, MI 48201, USA
[9]Center for Molecular Medicine and Genetics, Wayne State University School of Medicine, Detroit, MI 48201, USA
[10] Chimerix, Inc., Durham, NC, 27713

*Correspondence: gey@karmanos.org (Y.G.) or jtaub@med.wayne.edu

Running Title: Combined ONC213 and venetoclax in AML cells

Supplemental Methods

MTT Assay

MTT (3-[4,5-dimethyl-2-thiazolyl]-2,5-diphenyl-2H-tetrazolium bromide, Sigma-Aldrich) assays were performed as previously described [1, 2]. The cells were treated with various concentrations of venetoclax or a combination of venetoclax and azacitidine in a 1:3 ratio for 72 h. IC_{50} values are presented as mean values ± standard error of the mean (SEM) from at least three independent experiments, and those of the primary patient samples are presented as mean of duplicates from one experiment due to limited sample availability.

References

1. Zhao J, Niu X, Li X, Edwards H, Wang G, Wang Y, et al. Inhibition of CHK1 enhances cell death induced by the Bcl-2-selective inhibitor ABT-199 in acute myeloid leukemia cells. Oncotarget. 2016;7(23):34785-99.
2. Xie CZ, Edwards H, Xu XL, Zhou H, Buck SA, Stout ML, et al. Mechanisms of Synergistic Antileukemic Interactions between Valproic Acid and Cytarabine in Pediatric Acute Myeloid Leukemia. Clinical Cancer Research. 2010;16(22):5499-510.

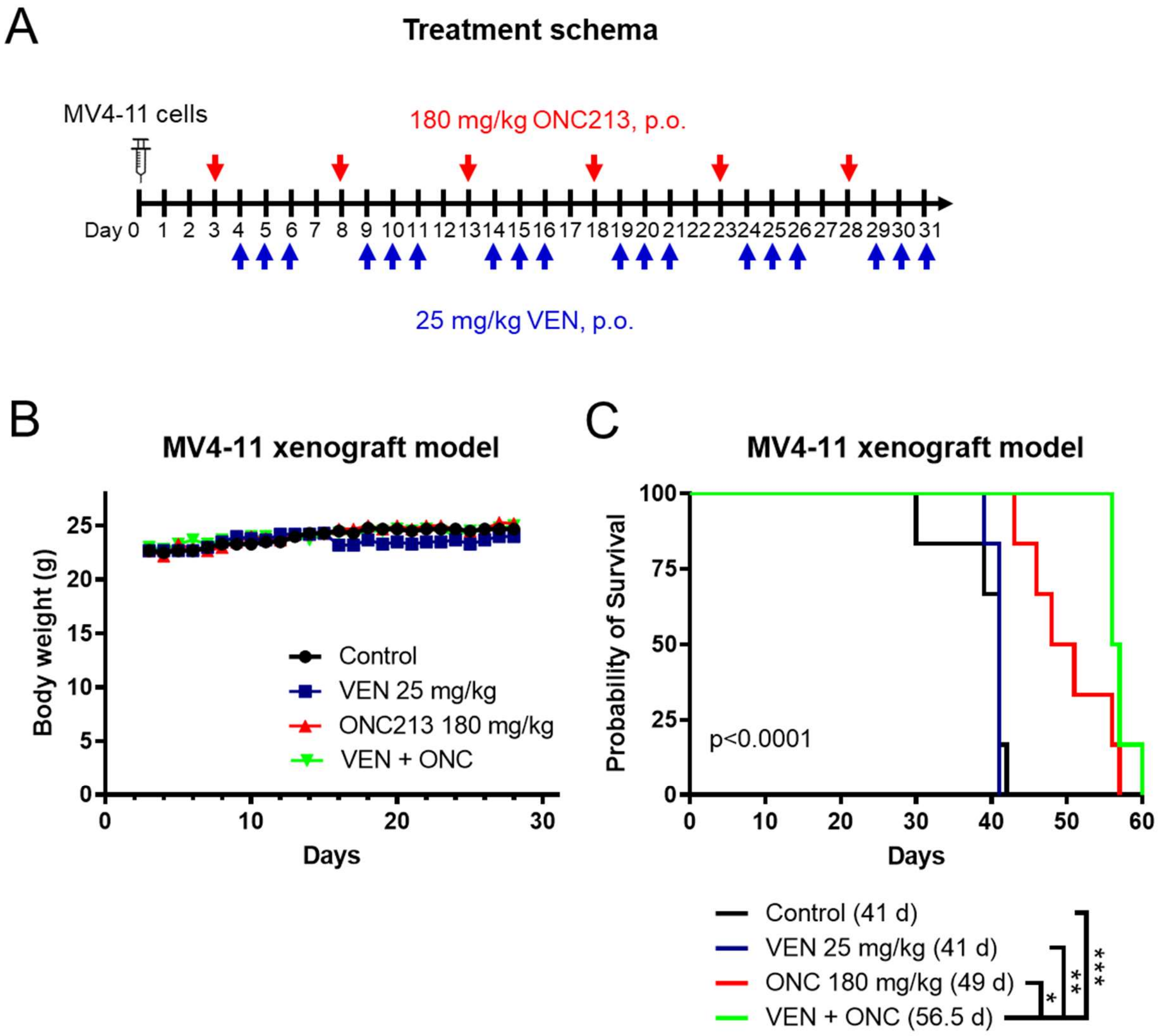

Figure S1. ONC213 + venetoclax has efficacy against AML cells *in vivo*. MV4-11 cells were injected into NSGS mice. On day 3, the mice were randomized into treatment arms (n=5) and treated as indicated in panel A. Mouse body weights are graphed in panel B. The overall survival probability was estimated using the Kaplan-Meier method (panel C). * $p<0.05$, ** $p<0.01$, and *** $p<0.001$.

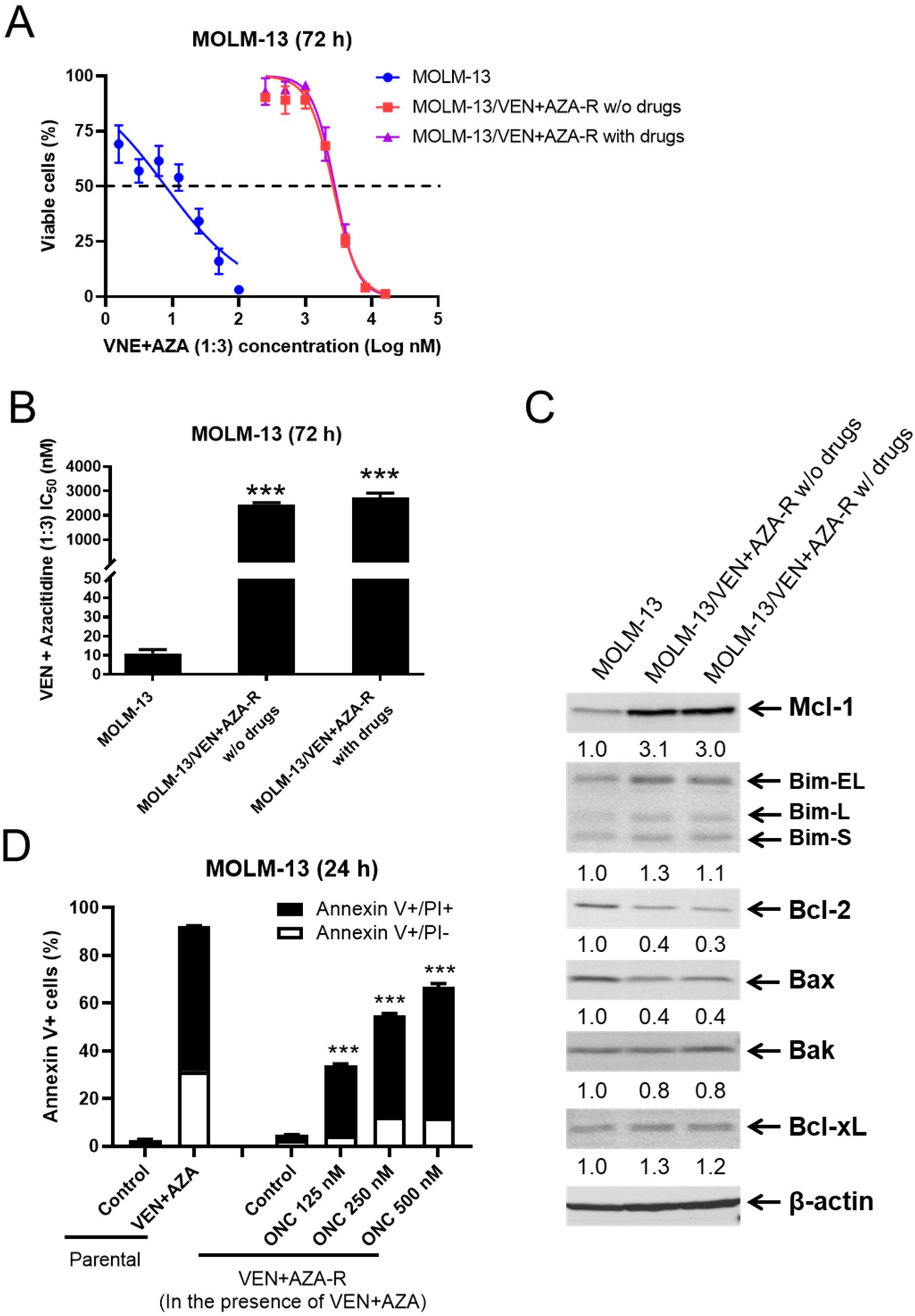
A
MOLM-13 (72 h)
MOLM-13
MOLM-13/VEN+AZA-R w/o drugs
MOLM-13/VEN+AZA-R with drugs
Viable cells (%)
VNE+AZA (1:3) concentration (Log nM)
B
MOLM-13 (72 h)
VEN + Azacitidine (1:3) IC50 (nM)
***
***
MOLM-13
MOLM-13/VEN+AZA-R w/o drugs
MOLM-13/VEN+AZA-R with drugs
C
MOLM-13
MOLM-13/VEN+AZA-R w/o drugs
MOLM-13/VEN+AZA-R w/ drugs
Mcl-1
1.0 3.1 3.0
Bim-EL
Bim-L
Bim-S
1.0 1.3 1.1
Bcl-2
1.0 0.4 0.3
Bax
1.0 0.4 0.4
Bak
1.0 0.8 0.8
Bcl-xL
1.0 1.3 1.2
β-actin
D
MOLM-13 (24 h)
Annexin V+/PI+
Annexin V+/PI-
Annexin V+ cells (%)
***
***
***
Control
VEN+AZA
Control
ONC 125 nM
ONC 250 nM
ONC 500 nM
Parental
VEN+AZA-R
(In the presence of VEN+AZA)

Figure S2. ONC213 + venetoclax resensitizes venetoclax+azacitidine-resistant cells to venetoclax. (A) MOLM-13/VEN+AZA-R cells were treated with increasing concentrations of VEN + AZA (fixed 1:3 ratio) for 72 h. MTT assays were performed. Viable cells relative to no drug treatment control is shown. (B) The IC_{50} values were calculated as the concentration of drugs that inhibit 50% of viable cells compared to no drug treatment control. *** $p<0.001$ compared to parental MOLM-13. (C) MOLM-13 and MOLM-13/VEN+AZA-R (with or without VEN + AZA in the media for 72 h prior to lysing the cells) whole cell lysates were subjected to western blot analyses. Representative western blots probed with the indicated antibodies are shown. The fold changes for the densitometry measurements, normalized to β-actin and then compared to parental MOLM-13 cells are indicated below the corresponding blots. (D) MOLM-13 cells treated with 1500 nM VEN + 4500 nM AZA and MOLM-13/VEN+AZA-R treated with ONC213 (ONC) for 24 h. Flow cytometry analyses of annexin V/PI staining is shown as mean ± sem. *** $p<0.001$ compared to control.

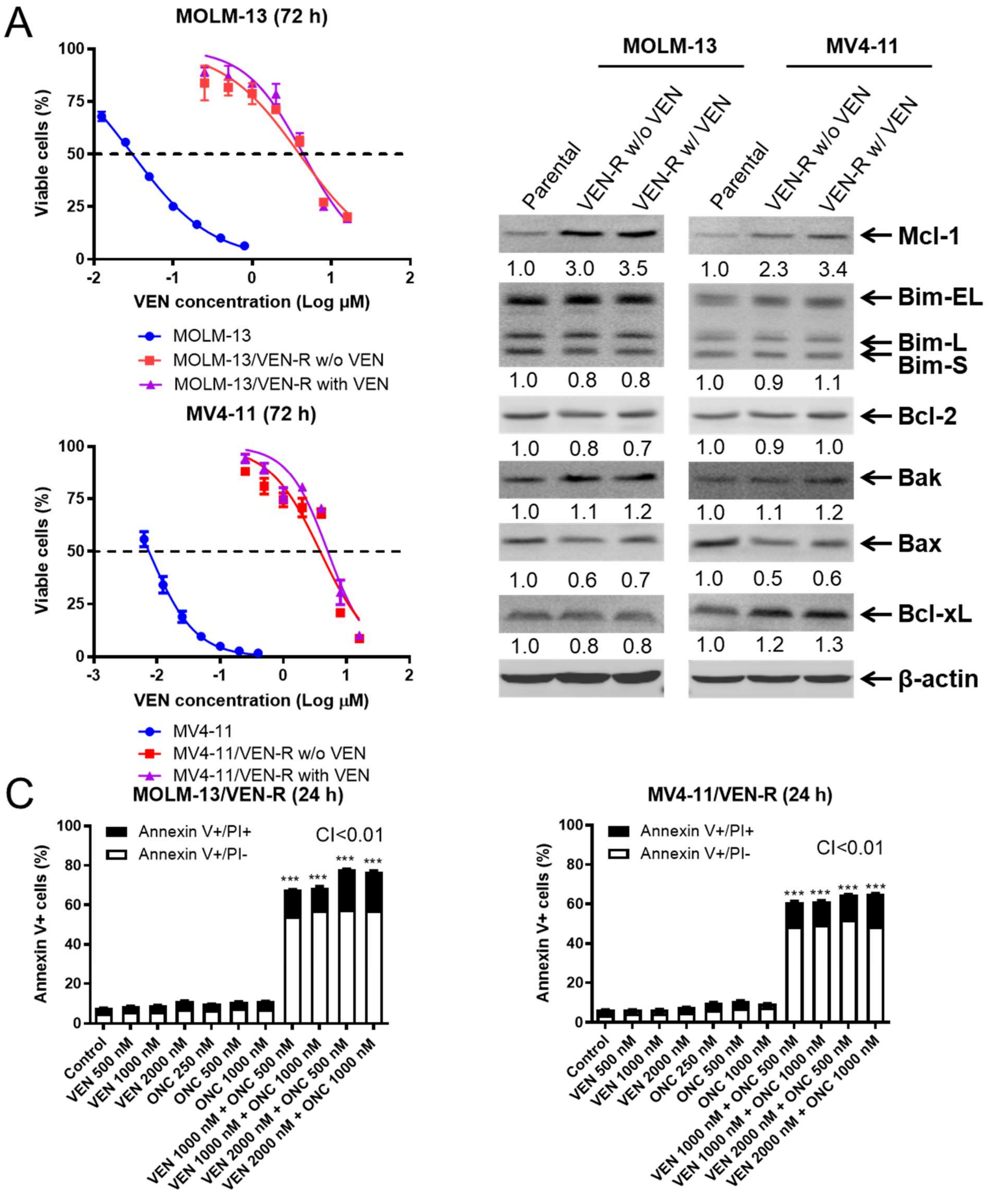

Figure S3. ONC213 + venetoclax resensitizes venetoclax-resistant cells to venetoclax. (A) MOLM-13/VEN-R and MV4-11/VEN-R cells were treated with variable concentrations of VEN for 72 h. MTT assays were performed. Percent of viable cells relative to no drug treatment control

is shown. (B) MOLM-13, MV4-11, MOLM-13/VEN-R, and MV4-11/VEN-R (cultured in the presence or absence of VEN in the media for 72 h prior to lysing the cells) whole cell lysates were subjected to western blot analyses. Representative western blots probed with the indicated antibodies are shown. The fold changes for the densitometry measurements, normalized to β-actin and then compared to parental cells are indicated below the corresponding blots. (C) MOLM-13/VEN-R and MV4-11/VEN-R cells were treated with VEN, ONC, or VEN + ONC for 24 h. Cells were then stained with annexin V/PI and subjected to flow cytometry analyses. Combination Index (CI) values were calculated using CompuSyn software. CI < 1.0, CI $= 1.0$, and CI > 1.0 indicate synergistic, additive, and antagonistic effects, respectively. *** $p<0.001$ compared to control and single drug treatments.

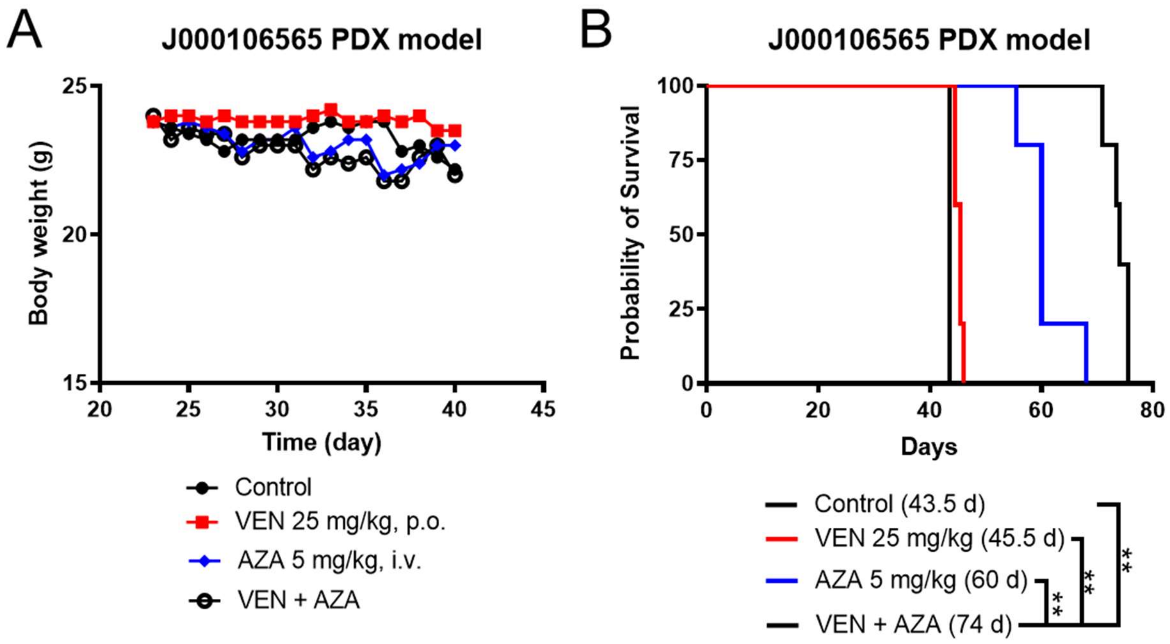

Figure S4. Venetoclax + azacitidine treatment of an AML PDX model. (A&B) J000106565 cells were injected intravenously through the tail vein of NSGS mice to generate a patient derived xenograft model. Human cell engraftment was verified in three randomly selected mice 22 days later by flow cytometry (average 20.23% hCD45+ cells in the peripheral blood). On day 23, the mice were randomized (n=5 mice/group) and treated as shown Figure 2D, upper panel. Mouse body weights are graphed in panel H. The overall survival probability was estimated using the Kaplan-Meier method (panel I). ** $p<0.01$.

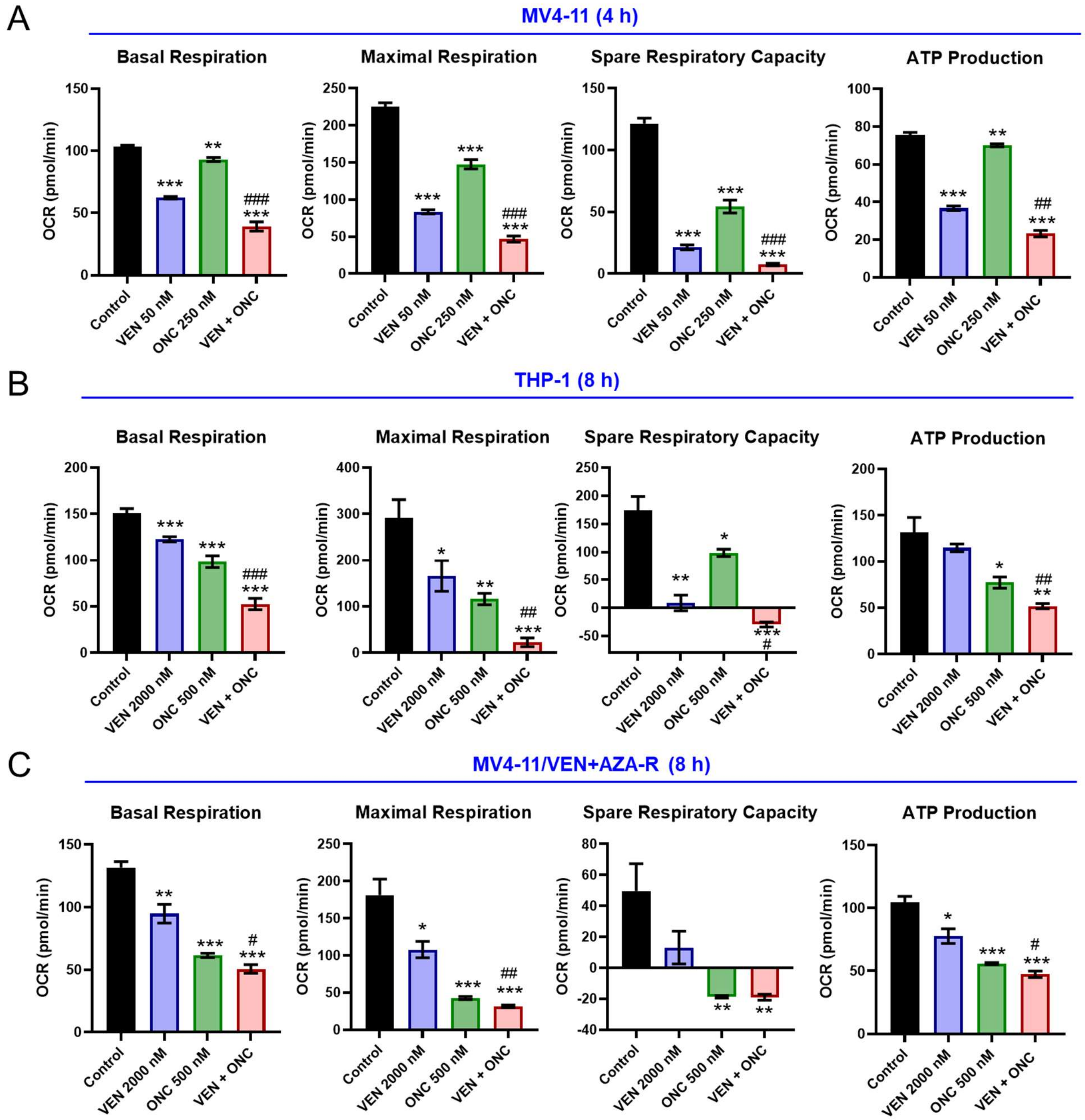

Figure S5. ONC213+venetoclax treatment decreases mitochondria respiration in AML cells. MV4-11 (panel A), THP-1 (panel B), and MV4-11/VEN+AZA-R (panel C) cells were treated with VEN and ONC, alone or in combination, for 4 or 8 h and then subjected to the cellular mito stress test (CMST). OCR was measured following injection with oligomycin A (complex V inhibitor), FCCP (mitochondrial membrane uncoupler), and rotenone + antimycin A (complex I and III

inhibitors). Basal respiration (baseline OCR – non-mitochondrial OCR), maximal respiration (maximal OCR following FCCP – non-mitochondrial OCR), spare respiratory capacity (maximal respiration – basal respiration) and ATP production (basal OCR – minimum OCR following oligomycin A) were determined via changes in OCR under CMST conditions are shown. * $p<0.05$, ** $p<0.01$, *** $p<0.001$ compared to control. ## $p<0.01$ and ### $p<0.001$ compared to ONC alone and VEN alone.

Table S1. Patient characteristics of primary AML patient samples

Patients	Gender	Age (year)	Disease status	FAB subtype	Cytogenetics	Blast purity (%)	Gene mutation
AML#193	Female	8	Newly diagnosed	M3	46, XX, t(15; 17)(q22; q21)	91.5	FLT3-ITD
AML#195	Male	7	Newly diagnosed	NA	46, XY	87.0	NA
AML#207	Female	66	Newly diagnosed	M3	46, XX, t(15; 17)(q24; q21)	92.0	FLT3-ITD/PML-RARα
AML#212	Male	11	Newly diagnosed	M2	NA	63.0	FLT3-ITD
AML#213	Male	3	Newly diagnosed	M4/5	46, XY	64.0	FLT3-ITD
AML#214	Male	8	Newly diagnosed	M2	46, XY	86.5	FLT3-ITD
AML#225	Male	28	Newly diagnosed	M4/5	46, XY inv(16)(p13q22)/46, XY	70.5	CBFB-MYH11, K-RAS
AML#226	Female	64	Newly diagnosed	AML	46, XX	87.0	CEBPA
AML#227	Male	32	Newly diagnosed	M4/5	47, XY,+14, inv(16)(p13q22)/46, XY, inv(16)(p13q22)/46, XY	87.5	PRAME, WT1, JAK2, NRAS

NA, not available
